# Editorial: We need to talk about authorship

**DOI:** 10.1093/gigascience/giy122

**Published:** 2018-11-02

**Authors:** Hans Zauner, Nicole A Nogoy, Scott C Edmunds, Hongling Zhou, Laurie Goodman

**Affiliations:** 1 BGI Hong Kong Ltd., 16 Dai Fu Street, Tai Po Industrial Estate, NT, Hong Kong SAR, China; 2 BGI Shenzhen, Bei Shan Industrial Zone, Yantian, Shenzhen, 518120, China

**Keywords:** authorship, ICMJE, Vancouver Convention, editorial policies, research misconduct

## Abstract

In our day-to-day editorial work at *GigaScience*, time and again we see issues cropping up that make us worry whether everyone understands good scientific practice when it comes to listing author names on the title page. There are many issues that underlie inappropriate authorship designations, but there are also guidelines to help potential authors determine when and how a researcher should be included with a manuscript. Here, we help clarify this and also provide a clear statement of our expectations around how authors are assigned to manuscripts submitted to *GigaScience*.

In publishing, authors and editors often run into problems related to making authorship decisions. Here, we hope to clear up some misunderstandings regarding who should be an author and who should take on special roles such as “first” and “corresponding” author. Because there is no reason to reinvent the wheel concerning defining authorship roles, *GigaScience* wholeheartedly embraces the excellent guidelines (also known as the Vancouver Convention or criteria) issued by the International Committee of Medical Journal Editors (ICMJE) [1], which we have adopted in our editorial policies (Fig. 1). We are also a member of the Committee on Publication Ethics (COPE), which has excellent discussion documents on what constitutes authorship and summarizes the (mostly consistent) practices of fields beyond biomedical research [2].

**Figure 1:**
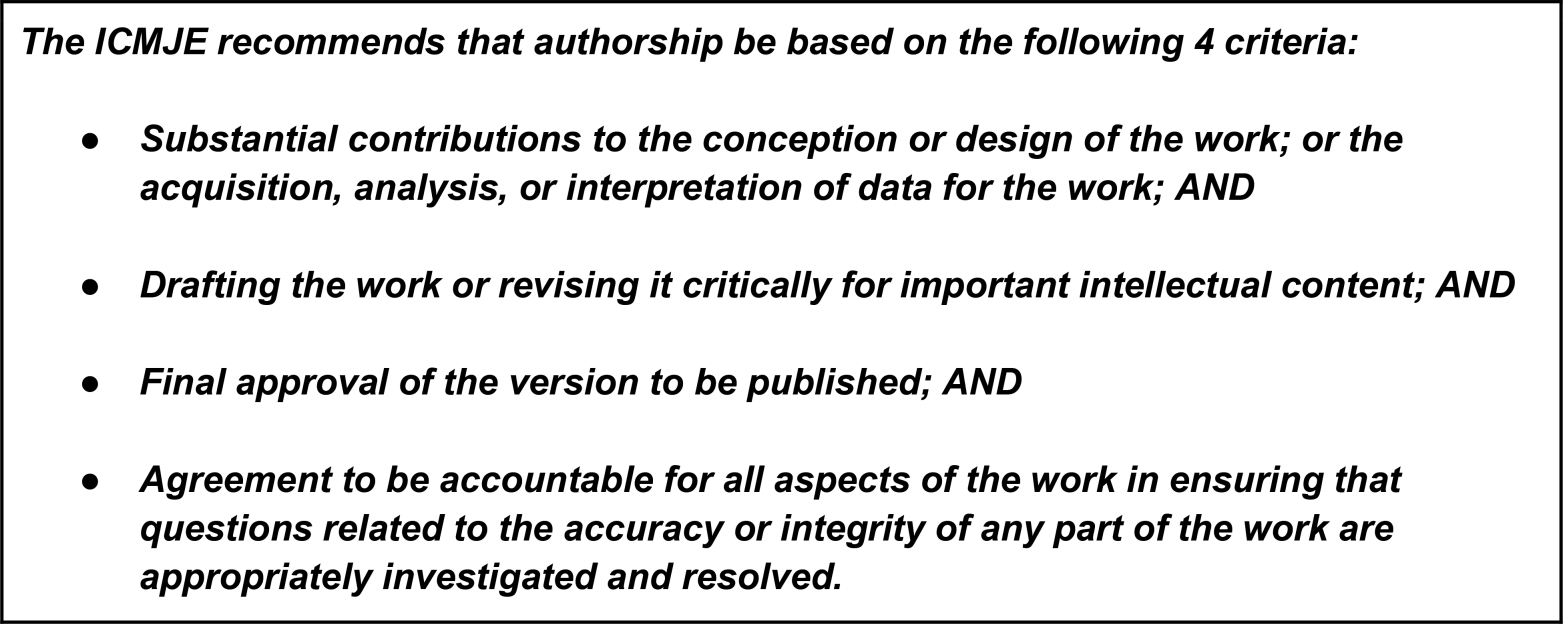
The four ICMJE/Vancouver criteria for authorship.

Getting authorship right is important. Being an author on research articles translates more or less directly into career and funding opportunities. Most biological and biomedical journals list authors according to an author-rated level of contribution: the first author is considered to have contributed more than the second author and so forth until reaching the author in the last position with that author usually being the “senior” author or “principal investigator.” Being or not being an author, and the order of that authorship, can make the difference between an upward career trajectory and one with fewer options. This is why rules matter. Getting authorship right is a matter of fairness. Therefore, we want to be as clear as possible on what is acceptable—and what is not.

Let's start at the beginning. Who is an author? An author is a person who creates a work, e.g., a novel, a movie, or a scientific research article. The latter, however, is a special beast. First, there is no unlimited creative freedom when writing up your scientific results, and a person can be an author even if they haven't provided any or very much of the actual writing. A research article involves a great deal more than conception and writing. Prior to even putting pen to paper, there are several phases that include planning, data gathering, analysis, and interpretation. Typically, many scientists, often with highly specialized expertise, work together during the lengthy course of a research project. Some very large collaborative projects can have more authors than words in the text. Case in point is a high energy physics article with more than 5,000 authors [3].With large consortia projects becoming more common in the life sciences, a genomics paper in 2015 had more than 1,000 authors [4]. It is worth noting, however, that the research community had some concerns about the inclusion of all of those authors—very much related to some of the issues we make note of here.

For research articles, an author is considered someone who has made an “intellectual contribution” to the work. Intellectual contributions include things like determining questions to investigate and deciding the main ways to do this, planning experiments, writing code, interpreting data, performing analyses, and so on. However, not all contributions are intellectual contributions, e.g., copy editing or providing samples and data that have already been used for a publication or where acquisition did not involve any input or understanding of the research project. Individuals making these latter types of contributions should be acknowledged in the Acknowledgement section of a manuscript.

## Who's on First? Joint First Authorship

There is no doubt that the pole position on the author list is special, as colleagues, grant agencies, and employers understand that the first author usually did most of the research and provided the greatest intellectual contribution, e.g., as part of a PhD thesis. Given the special significance of the first author, it is understandable that for some authors it is tricky to decide who should go first, especially for projects that require distinctly different areas of expertise to complete the work. Given this, it can be appropriate to indicate that two or, in rare cases, three authors contributed in equal measure to a manuscript. It is particularly important for PhD students and junior researchers to get this credit in order to progress in their careers, and it is difficult to not get lost in the crowd for large consortia projects. However, the likelihood that shared first authorship is warranted seems to decrease with the number of joint first authors. We have recently seen up to 11 joint first authors listed on the title page of submitted manuscripts. We could not decide, especially given that this was not a project that required numerous different areas of expertise, how it was possible for them to all have done exactly the same amount of work. In light of increasing first author inflation,*GigaScience* now allows a maximum of three joint first authors, and their roles must be made clear and justification must be given as to how they have carried out equivalent amounts of intellectual contribution.

In medicine there has been much talk of moving from co-authorship to contributorship, which allows readers to more accurately assess credit and accountability [5]. We strongly agree with this. In addition to asking for contributorship details in an Author Contributions section, we also collect Consortia Advancing Standards in Research Administration Information (CASRAI) contributorship taxonomy information in the submission process. We also provide authors the option to include an Authors' Information section for additional context on who did what. With our previous publisher, we experimented presenting this via Mozilla badges. While we are now collecting this information on submission, we are looking for ways to better present this information to our readers. You can see in the Author Contributions section of this editorial how this information can be displayed in the form of a list.

## Confusing “Corresponding Author” with Author Seniority

The corresponding author is not necessarily the most senior author or project lead. There is a special responsibility involved when taking on this role, but being a corresponding author is not supposed to be a mark of distinction. Corresponding authors, for the most part, act as a secretary. They are the primary contact for the journal and respond to all manuscript queries. They should also be able to provide details on authorship, contributions, and ethics approval and to gather conflict of interest statements. If the article is published, corresponding authors need to respond to any questions that readers or editors might have. Often, the most senior author is too busy to reply to queries during the submission and review process, and papers have been delayed in the publication process as a result, sometimes for months. If corresponding authors are doing a poor job of being responsive before a paper has even has been published, how likely are they to respond to reader comments and queries many years in the future? Unresponsiveness at the start is a clear sign that this individual should not have taken on this task from the start. We suggest choosing the most suitable author (or authors) who has the time to contact all the other authors, can explain the vast majority of the research in detail, and can respond in a timely manner.

**Figure 2:**
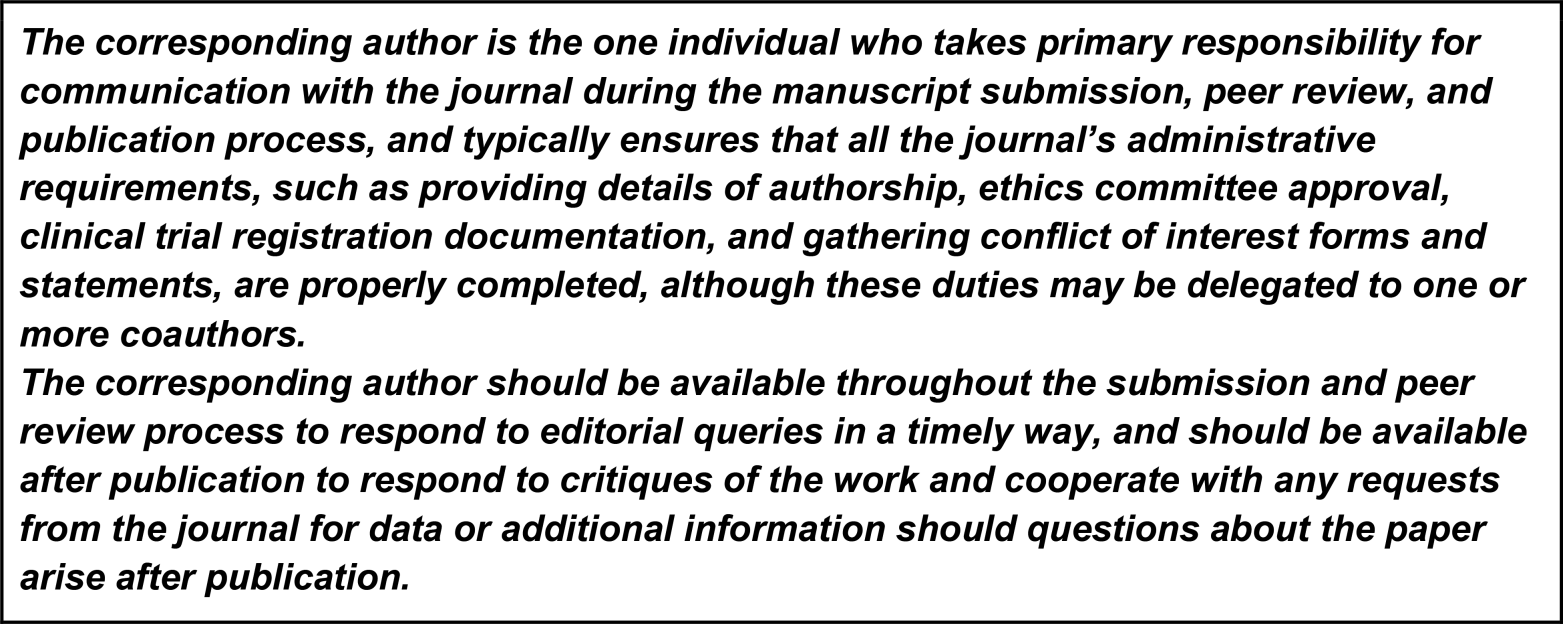
ICMJE/Vancouver guidelines on the role of corresponding authors.

Sometimes, it is reasonable that two authors share the role of corresponding author. For example, if two groups collaborate on a project, it can make sense that there is a primary contact for each group. It is also useful to have multiple contact details to share the administrative load. However, similar to joint first authors, we have seen too many submissions where designations of three or even four “joint” corresponding authors worry us (Fig. 2.).

## Authorship as a Commodity

Gift authorship to curry favor and ghost authorship from the pharma industry are long running phenomena, but a more industrialized version of this has cropped up in recent years: “paper mills.” In addition to an underground “academic bazaar” where authorship can be bought [6], a systematic network of Chinese companies that produce ghostwritten papers to order has been exposed. The recent mass retractions of papers from a number of publishers linked to the hacking of the peer-review system is a direct side effect of this [7]. This large-scale gaming of the system has been driven by skewed incentive systems, where authorship of papers in Science Citation Index (SCI)-indexed journals is worth huge amounts of money. Chinese universities offer cash rewards of up to $165,000 (USD) for papers published in the top SCI-indexed journals [8]. Medics there are required to publish in impact factor journals for promotion. To feed this requirement, companies have been caught offering ghostwritten publications in impact factor 1-2 journals for $10,000 (USD) [9]. As some Chinese funders are specifying that first and corresponding authors are the ones who get cash rewards [10], this misunderstanding of international norms on authorship is likely one of the drivers of the joint authorship inflation we've been seeing, and greater awareness of the ICMJE guidelines could help to address this.

## After Acceptance Authorship

Misunderstanding who should be an author can create complex situations in the publishing process. One thing that has gotten very complicated is requests to add authors after article publication. There are many reasons for these requests, some because there is a very an extremely large number of individuals involved in the research, and some people are accidentally left off the list. However, there is a growing issue regarding individuals being added to manuscripts for less benign reasons, often financial. Adding (or removing) authors at the last minute, especially because of some unethical practices, can create large delays as editors have to assess the reasons for additions and contact individuals who have been removed. Please think carefully about who should be an author and use the ICMJE guidelines when doing this. At *GigaScience*, we have become very stringent about author additions after acceptance and require detailed reasons for why they need to be added. There have been cases where we declined to add them.

So, before submitting, ask yourself who in your group or in other groups collaborated during the project? Get in touch with all potential authors and discuss authorship *early* in the writing process. Assess the level at which individuals outside of the main groups contributed to the project. If it does not fit with any of the detail in the ICMJE guidelines, do not include them. However, discuss it with them nevertheless. Before submission—do not just add names to the author list without mentioning it in the cover letter, perhaps hoping we won't ask questions. We will do that!

Our rules and lessons learned are summarized above and in Box 1. We expect our authors to fight the misguided temptations, attitudes, and incentives attached to the roles of corresponding and first author. As the first and corresponding authors are formally highlighted with an asterisk and dagger footnotes, one short-term fix to take the pressure off the corresponding authors could be to highlight senior authors in a similar manner (e.g., with a double dagger ‡, as demonstrated in this editorial). The move toward contributorship rather than co-authorship will remove much of these pressures and skewed incentive systems, but until then, there has to be education and enforcement of best practices. In addition to the coordinating moves of ICMJE, COPE, CASRAI, and others, we hope we can all work together to address these matters. We hope this editorial has addressed the misinterpretation of what authorship and corresponding authorship relates to. Policy, procedure, and publishing infrastructure continue to evolve, and we welcome feedback from our authors on how this affects them and how we can continue to improve the process.

**Box 1:** Lessons learned and take-home recommendations for authorship

*GigaScience* allows a maximum of three joint first authors and two corresponding authors. Please think carefully as to who actually contributed (as per ICMJE guidelines) and can handle these roles. See our Author Guidelines (https://academic.oup.com/gigascience/pages/authorship_guidelines). The corresponding author is not the senior/supervisory author. It must be someone who can respond to all manuscript queries in a timely manner.Finalize your author list *before* submission—we only consider additional authors if extensive revision (e.g., more experiments) is required after peer review.Contributorship rather than co-authorship:● Upon submission, provide as much detail as you can when completing the CASRAI authors roles upon submission. You can also include this information in the Author Contributions section.● Provide additional context on who did what. You can also include an Authors' Information section with additional context on who did what.Provide more granular and precise credit via data, software, and method/protocol citations.Cash rewards based on authorship and focus on one-dimensional journal-based metrics, such as SCI ranking, distorts science and encourages gaming of the system and fraud.Like us, we encourage people to sign DORA (the Declaration on Research Assessment [https://sfdora.org/]) and to stop using journal-based metrics as a surrogate measure of the quality of research articles or authorship in them to assess an individual scientist's contributions.

## Additional files

Supplemental File 1: Extended Chinese language (中文版) version on the editorial.

## Abbreviations

CASRAI: Consortia Advancing Standards in Research Administration Information; COPE: Committee on Publication Ethics; DORA: Declaration on Research Assessment; ICMJE: International Committee of Medical Journal Editors; SCI: Science Citation Index; USD: United States Dollars.

## Author contributions

Writing original draft: H.Z., N.A.N., S.C.E., L.G.; investigation: H.-L.Z.; review and editing: H.Z., N.A.N., S.C.E., H.-L.Z., L.G.; supervision: L.G.; conceptualization: H.Z., N.A.N., S.C.E.

## Supplementary Material

Supplemental File
