# Supplementary material for: Editorial: We need to talk about authorship
Source: Gigascience. 2018 Nov 2;7(12):giy122. doi: 10.1093/gigascience/giy122 (PMC6283212; doi:10.1093/gigascience/giy122)
Supplement: Supplemental File [file giy122_supplemental_file.docx]

**规范作者署名问题刻不容缓**

Hans Zauner^1^ Nicole A Nogoy^1^ Scott C Edmunds^1,2^ 周红玲^2^* Laurie Goodman^1, 2^‡

^1^（*GigaScience* 香港）

^2^（深圳华大生命科学研究院 深圳 518120）

*通讯作者：周红玲, [hongling@gigasciencejournal.com](mailto:hongling@gigasciencejournal.com), ORCID: 0000-0002-7295-8176

‡高级作者：Laurie Goodman, [laurie@gigasciencejournal.com](mailto:laurie@gigasciencejournal.com), ORCID: 0000-0001-9724-5976

**摘要：**在*GigaScience*期刊的日常工作中，与作者署名相关的问题屡见不鲜，这让我们担忧作者们是否了解署名时需要遵守的科学规范。大多数问题集中在不恰当的作者署名，但其实有一些指导原则可以帮助投稿人决定何时以及如何将研究者列为作者。本文拟帮助明确这些原则，同时也明确了*GigaScience*对作者署名方面的期望。随着基金资助者和政策制定者对有关科研评价和科研诚信政策的出台，对这些政策如何与科学实践保持一致并促进科学实践，我们也提出了相应见解。

**关键词：**作者署名，ICMJE，编辑政策，科研不端，作者贡献

**分类号：**G312

**We need to talk about authorship**

Hans Zauner^1^ Nicole A Nogoy^1^ Scott C Edmunds^1,2^  Zhou Hongling^2^* Laurie Goodman^1, 2^‡

^1^ (*GigaScience*, Hong Kong, China)

^2^ (BGI Shenzhen, Shenzhen, 518120, China)

*corresponding author: Zhou Hongling, [hongling@gigasciencejournal.com](mailto:hongling@gigasciencejournal.com), ORCID: 0000-0002-7295-8176

‡ Senior author: Laurie Goodman, [laurie@gigasciencejournal.com](mailto:laurie@gigasciencejournal.com), ORCID: 0000-0001-9724-5976

**Abstract:** In our day-to-day work running *GigaScience* journal, time and again we see issues cropping up that make us worry whether everyone understands good scientific practice when it comes to listing author names on the title page. There are many issues that underlie inappropriate authorship designations, but there are also guidelines to help potential authors determine when and how a researcher should be placed on a manuscript. Here we help clarify this, and also provide a clear statement of our expectations around how authors are assigned to manuscripts submitted to *GigaScience.* With funders and policy makers updating their procedures regarding research evaluation and credit, we also provide our perspective on how these need to be consistent and promote good practice.

**Keywords:** Authorship, ICMJE, Editorial Policies, Research Misconduct, Contributorship

**正文：**

在出版过程中，作者和编辑们经常遇到与决定作者身份相关的问题。本文希望澄清相关误解，比如谁应该是作者，以及谁应该作为“第一”作者和“通讯”作者等特殊角色。目前已经有大量用于定义作者角色的信息，对此，*GigaScience*全然接受国际医学期刊编辑委员会（全称，ICMJE）发布的优秀指导原则^1^。本刊与作者署名相关的编辑政策便采用了这些指导原则（图1）。同时，我们也是国际出版伦理委员会（COPE）成员，该委员会有非常好的关于作者署名的讨论文件，并总结了生物医学研究之外的其他领域（多数情况下是一致的）规范^2^。

正确署名作者十分重要。在某种程度上，成为科研论文的作者可直接与事业和基金资助机会挂钩。大多数生物学和生物医学期刊根据作者贡献度依次列出作者：认为第一作者的贡献高于第二作者，依此类推，最后一位作者通常是“高级作者”或“主要研究者”。是否被列为作者以及作者署名次序，可以决定研究人员事业上升还是受阻。

这也就是作者身份如此重要的原因。正确的作者署名关乎公平。因此，我们希望尽可能明确什么是可接受的，什么是不可接受的。

第一步：谁是作者？作者是创建作品的人，例如，一本小说、一部电影或一篇科研文章；但后者比较特殊。首先，在撰写科研成果时没有无限的创作自由，即使没有参与文章的任何实际撰写，一个人也可以成为作者。一篇科研文章的内容远远超越了概念和写作。撰写文章之前有数个阶段，包括研究计划、数据收集、分析和解释。通常一个漫长的研究项目由许多具有较高专业知识水平的科学家合作完成。因此，某些大型合作项目的文章甚至可能出现作者篇幅超过正文的情况：比如一篇高能物理学文章有超过5000位作者^3^。随着生命科学领域内的大型合作项目越来越普遍，2015年发表的一篇基因组学文章有超过1000位作者^4^。然而，值得注意的是科研界对于列入所有这些作者存在一些担忧，这与我们在这里提到的一些问题密切相关。

在科研文章中，作者被认为是对研究工作做出“智力贡献”的人。智力贡献包括但不限于以下内容：设定科学问题并决定解决该问题的主要方法、实验规划、代码编写、数据解释、分析执行等等。但是并非所有的贡献都是智力贡献，比如校样、修改或提供已发表文章中使用的样本和数据，或者不涉及对该研究项目的任何投入或理解的样本和数据获取。此时应该在文章的“致谢”部分列出并感谢做出后一类贡献的人。

| **国际医学期刊编辑委员会建议根据以下四条标准确定作者身份：**   - **对研究工作的思路或设计有实质贡献；或者为研究获取、分析或解释数据；且** - **起草论文或在重要的智力性内容上进行了关键性修改；且** - **对将要发表的版本作最终定稿；且** - **愿意对研究工作的各个方面承担责任，以确保与研究工作任何部分的准确性或诚信相关的问题得到妥善调查和解决。** |
| --- |

**图1** **国际医学期刊编辑委员会确定作者身份的四条标准**

**谁是第一作者？共同第一作者**

毫无疑问，在作者名单中第一位是特殊的，因为同行、资助机构和雇主都了解，第一作者通常做了大部分研究并提供了最多的智力贡献，例如，博士论文。鉴于第一作者的特殊意义，可以理解有时很难决定谁应该是第一作者，尤其是对于需要不同领域专业知识才能完成的项目。鉴于此，可以指定两个或三个（在极少数情况下）同等贡献作者。对于博士生和初级研究者来说，获得这种荣誉对于他们职业生涯的进步尤其重要，同时这类研究者也很难在大型合作项目的众多研究者中脱颖而出。然而，共享第一作者身份的可能性似乎随着共同第一作者的数量增加而下降。我们在最近投稿的文章中看到了多达11位共同第一作者，我们无法确定这11位共同第一作者如何完成了等量的工作，尤其这不是一个需要大量不同领域专业知识的项目。鉴于第一作者的数量与日俱增，*GigaScience*目前最多允许三位共同第一作者，且必须指明他们的角色，并说明他们如何完成了等量的智力贡献。

为了平衡对第一作者数量限制的影响，我们鼓励更好地利用“引用率”来体现更多点滴的贡献。这就是为什么我们通过Data Note文章来认可数据生产者的贡献，通过Technical Note文章认可软件开发者的贡献。此外，通过单独引用和认可对整体工作做出贡献的、一篇文章的特定组成部分（如数据、计算机源代码、协议和工作流等）（上述每个组件均被称为“研究对象”），从而使贡献更加细化。为了直接引用研究对象，我们在“GigaDB数据库”或其他平台（如protocols.io）中为这些研究对象分配DOI号，这使各类工作人员的贡献可以通过数据引用、软件引用和方法引用得到认可。

在医学方面，有很多关于从“共同作者（Co-authorship）”向“作者贡献（Contributorship）”转变的讨论，这一转变可使读者能够更准确地评估作者的信誉和责任^6^。我们非常赞同这一点，除了在作者贡献部分包含每位作者所做的工作内容之外，我们目前还使用CASRAI贡献分类法的正式方式在投稿过程中收集这一信息^7^。越来越多的出版商（包括eLife、Cell Press和PLOS）采用了这种方法，而美国国家科学院也一直在鼓励基金资助者接受CRediT分类法^8^。我们还为作者提供了一个选项，即附加“作者信息”部分，以便更清晰地了解谁做了什么。在与以前的出版商合作期间，我们尝试通过Mozilla badges进行展示，目前我们在作者投稿时收集这些信息，也正在寻求更好的呈现该信息的方式，例如以列表的形式呈现。

**混淆“通讯作者”与作者资历**

通讯作者不一定是资历最高的作者或项目负责人。通讯作者需要承担特殊的责任，但不应该成为特殊荣誉的标志。通讯作者大部分情况下是担任秘书角色。其作为期刊的主要联系人，负责回应稿件相关的所有疑问；还应能够提供作者和贡献方面的详细信息、伦理批准，并收集利益冲突声明。如果文章发表，通讯作者需立即回复读者或编辑的任何疑问。在投稿和审稿过程中，资历最高的作者通常因太忙而无法回复问题，我们在出版过程中也遇到过此类原因导致文章延迟发表的情况，有时可延迟数月。如果通讯作者在文章发表之前回应度很差，那么他们在未来几年回应读者的评论和疑问的可能性又有多大呢？最开始的无回应是一个明确的信号，即他们一开始就不应该担任通讯作者。我们建议选择最合适的作者或有时间联系所有其他作者的作者，保证其可以详细解释绝大多数研究，并及时做出回应。

有时由两位作者共同担任“通讯作者”是合理的。例如，如果两个团队合作完成一个项目，则每个团队各有一位主要联系人是说得通的。并且有多个联系人共同分担管理任务也很有帮助。然而与共同第一作者类似，我们看到太多稿件指定了三个甚至四个“共同”通讯作者，这让我们十分担忧（图2）。

| **通讯作者是在文章投稿、同行评议及出版过程中主要负责与期刊联系的人，并确保所投稿件达到期刊的所有管理要求（例如提供作者署名的详细信息、伦理委员会审核批件、临床试验注册文件，收集利益冲突表格和声明），虽然这些任务可能分配给一位或几位共同作者。**  **通讯作者应在整个投稿和同行评议过程中及时回复编辑方面的问题，并应在论文发表后及时回复对该研究工作的评论，如果文章发表后出现文章相关的问题，应配合期刊提交任何需要的数据或其他信息。** |
| --- |

**图2 国际医学期刊编辑委员会对通讯作者身份确认的指导原则**

**作者署名商品化**

将作者署名作为礼物馈赠以及制药业的代笔作者是长期存在的现象，但近年来演变得更为工业化：“论文工厂”。除了可以购买作者身份的地​​下“学术集市”之外^9^，中国公司应要求代写论文的系统化网络已被披露。最近，许多出版商大量撤销了与黑客入侵同行评议系统有关的文章，就是“论文工厂”的直接副作用^10^。这种系统的大规模操纵机制是由歪曲的激励系统驱动的，即在 SCI（科学引用索引）收录期刊上发表文章的作者身份价值不菲。中国大学为在顶级SCI收录期刊上发表的论文给予高达165,000美元的现金奖励^11^。虽然中国的医生非常忙碌，但经常被要求在有影响因子的期刊上发表文章用于职位晋升，为了满足这一要求，已经发现有公司在影响因子1-2的期刊上、以10,000 美元的价格提供代写文章服务^12^。

尽管中国已经尝试解释作者署名规范^13^，但这些都与基金资助者鼓励的指导方针和做法相矛盾，作者得到的最明确信息是第一作者和通讯作者可以获得SCI相关的现金奖励。即使是最有理想的科学家，也可能会被这些物质条件所诱惑。来自中国科学院某研究所的内部文件表明了现金奖励是如何分配的，原则上第一作者可获得40%的现金奖励，通讯作者大约为30%，非第一作者或通讯作者为10%。又例如，深圳2017年更新的“国家级领军人才”和“孔雀计划”海外高层次人才奖励政策中，在*Nature*或*Science*上发表论文的第一作者和通讯作者可获得最高三百万人民币的现金奖励^14^。由于*GigaScience*属于“JCR Q1”期刊，因此在“孔雀计划”海外高层次人才奖励政策下，作者仍可获得大约一半的现金奖励，这显然导致了我们和其他期刊所面对的巨大“共同作者通胀”压力。

这些有失偏颇的激励措施可能会导致腐败行为的发生，我们曾在香港媒体上发表了Op-Eds，指出导致中国大陆科研丑闻的相同奖励制度可能在香港导致同样的问题^15^。写完这篇文章后不久，我们发现一家香港注册公司（很欣慰现在已经消失）在我们伪装成研究者时向我们提供了“保证影响因子2”的文章发表服务，价格约8,000美元。虽然这种类型的系统性造假很可能相对罕见，但对所有学者和医生的“不发文章就走人”的要求使他/她们的压力与日俱增：即你如何才能被列为作者的压力。所有这些都可能与作者身份的“国际准则”和“ICMJE指导原则”不相符。值得注意的是，我们现在看到政府正在采取措施来解决这些问题，但基金管理部门和当地资助者必须对其资助程序作出非常具体的改变，以进一步减少这些问题，并需要在上述方面做出明确表态。

**在文章接受后变更作者署名**

在出版过程中，对于谁应该成为作者的误解可能会造成更多复杂的情况。在文章发表后要求添加作者就是一件非常复杂的事情。这些要求有诸多原因，部分原因是参与研究的人数非常多，有些人不小心被忽略在名单之外。然而，越来越多的添加作者的原因并非出于善意，通常是财政原因^9^。在最后一刻添加（或删除）作者，特别是出于某些不道德的原因，可能会造成很长时间的发表延迟，因为编辑必须评估添加作者的原因，并联系被删除的作者。请大家仔细考虑谁应该成为作者，并在决定作者身份时遵守ICMJE指导原则。*GigaScience*对在文章接受之后添加作者非常严格，要求详细说明他们需要被添加为作者的原因，某些情况下会拒绝添加作者。

因此，在投稿之前，一定要问自己，在你的团队中或其他团队中有谁参与了该项目合作？ 在文章撰写**初期**就应该与所有可能作者取得联系并讨论作者身份，评估主要团队以外的个人对项目的贡献水平。 如果不符合ICMJE指南中的条款，就不要将其列为作者；即便不将其列为作者，也要与他们讨论决定。在投稿之前不仅要在作者列表里添加作者名字，还应在投稿信（cover letter）中提及，或许大家寄希望于我们不会过问—但我们会的！

| ***GigaScience*允许至多3位共同第一作者和2位通讯作者。—请考虑清楚谁做出了实际贡献（根据ICMJE指南），并能够担任这些角色。我们的作者指南请见如下链接：** [**https://academic.oup.com/gigascience/pages/authorship_guidelines**](https://academic.oup.com/gigascience/pages/authorship_guidelines)  **通讯作者 —不是高级别作者 / 管理者。通讯作者必须是可以及时回应所有文章相关疑问的人。**  **在投稿前最终确定作者列表 —我们只在同行评审后需要大修（例如，增加实验）的情况下考虑增加其他作者。**  **提供作者贡献（Contributorship）而不是共同作者身份（co-authorship）—**   - **投稿时，在填写CASRAI作者角色时，应尽可能提供详细信息，还可以在“作者贡献”部分包含这些信息。** - **提供关于谁做了什么的额外背景信息，还可以在“作者信息”部分纳入这些信息。**   **通过引用数据、软件和研究方法 /步骤提供更细化和精确的作者分工信息。**  **基于作者身份的现金奖励和对期刊单一维度指标（如SCI排名）的过度重视会扭曲科学，并鼓励孤注一掷和学术造假。**  **我们鼓励大家签署DORA（研究评估声明，<https://sfdora.org/>），并停止使用期刊指标作为科研文章质量或文章中作者身份的替代指标来评估每个科学家的贡献。** |
| --- |

**图3 经验教训和确定作者身份的建议**

图3总结了我们的原则和经验教训。目前这些规则已经清晰详尽，并解释了我们为什么要做这些事，否则会造成巨大的问题，我们期望作者能够抵制这些与通讯作者和第一作者挂钩的误导性诱惑、态度和激励制度。

**结语：**

可将第一作者和通讯作者用星号（*）和剑号（†）脚注的形式突出显示，短期内可以通过类似的方式突出显示高级作者（例如用双剑号‡，如本篇社论所示）来减轻通讯作者的压力。向“作者贡献（contributorship）”而非“共同作者（co-authorship）”的转变将消除大部分压力和歪曲的激励体系，但在整个出版界完全实施该规则之前，必须向研究者宣传和普及这种最佳实践方式。除了ICMJE、COPE、CASRAI和其他团体的协调举措之外，我们希望大家可以共同努力解决这些问题。我们希望本文可以帮助解决对作者身份和通讯作者身份与政策、程序和出版机构相关内容不断演变的误解。令人鼓舞的是，科技部最近发布了新的改革措施，以解决中国的学术不端行为^16,17^。文件指出，未来将会重点关注深化科研评价体系改革，我们希望这一举措可以禁止不良做法，如SCI相关的现金奖励。一个更具深度的评价系统，需要使评价流程超越共同作者和影响因子之上，推广符合ICMJE指南的国际规范。我们期待看到这些新规将如何实施，除了协助教育并提出我们对该问题的看法之外，我们欢迎读者对这如何影响作者，以及我们如何继续改进评价过程提供反馈意见。

**作者贡献声明**

初稿撰写：Hans Zauner，Nicole A Nogoy，Scott C Edmunds， Laurie Goodman

前期调研：周红玲

审核编辑：Hans Zauner，Nicole A Nogoy，Scott C Edmunds，周红玲，Laurie Goodman

文章指导：Laurie Goodman

抽象概括：Hans Zauner，Nicole A Nogoy，Scott C Edmunds

**致谢**

感谢来自OUP的Julia McDonnell的反馈意见。

**缩略词**

CASRAI：推进研究管理信息标准联盟；COPE：出版伦理委员会；DORA：科研评估宣言；ICMJE：国际医学期刊编辑委员会；SCI：科学引用索引；USD：美元。

**参考文献**

[1] ICMJE. Defining the Role of Authors and Contributors.

http://www.icmje.org/recommendations/browse/roles-and-responsibilities/defining-the-role-of-authors-and-contributors.html

[2] COPE. What constitutes authorship? COPE Discussion Document. (2014).

https://publicationethics.org/files/Authorship_DiscussionDocument.pdf

[3] Aad, G. et al. Combined Measurement of the Higgs Boson Mass in. Phys. Rev. Lett. 114, 191803 (2015).

[4] Leung, W. et al. Drosophila muller f elements maintain a distinct set of genomic properties over 40 million years of evolution. G3 (Bethesda). 5, 719–40 (2015).

[5] Sneddon, T. P. et al. GigaDB: promoting data dissemination and reproducibility. Database 2014, bau018-bau018 (2014).

[6] Baerlocher, M. O., Newton, M., Gautam, T., Tomlinson, G. & Detsky, A. S. The meaning of author order in medical research. J. Investig. Med. 55, 174–80 (2007).

[7] CRediT Working Group. CRediT (Contributor Roles Taxonomy) website. http://docs.casrai.org/CRediT

[8] Mcnutt, M. K. et al. Transparency in authors’ contributions and responsibilities to promote integrity in scientific publication. doi:10.1073/pnas.1715374115

[9] Hvistendahl, M. China’s publication bazaar. Science 342, 1035–9 (2013).

[10] Seife, C. For Sale: ‘Your Name Here’ in a Prestigious Science Journal. Scientific American (2014).

http://www.scientificamerican.com/article/for-sale-your-name-here-in-a-prestigious-science-journal/

[11] Quan, W., Chen, B. & Shu, F. Publish or impoverish: An investigation of the monetary reward system of science in China (1999-2016). (2017). doi:10.1108/AJIM-01-2017-0014

[12] Filion, G. A flurry of copycats on PubMed. The Grand Locus blog (2014).

http://blog.thegrandlocus.com/2014/10/a-flurry-of-copycats-on-pubmed

[13] 贾贤, 王霞, 李忠富 & 佟建国. 科技论文中等同贡献作者和共同通讯作者的署名问题. 中 国 科 技 期 刊 研 究 24, (2012).

[14] Shenzhen Bureau of Human Resources and Social Security. 高层次专业人才管理 - Peacock Program for High-level professional talent management. (2017).

http://www.szhrss.gov.cn/xxgk/zcfgjjd/gcjzyrcgl/201708/t20170831_8317284.htm

[15] Edmunds, S. C. & Davidson, R. L. China must restructure its academic incentives to curb research fraud. South China Morning Post (2015). http://www.scmp.com/comment/insight-opinion/article/1758662/china-must-restructure-its-academic-incentives-curb-research

[16] Cyranoski, D. China introduces sweeping reforms to crack down on academic misconduct. Nat. 2018 5587709 (2018).

[17] MOST. 中共中央办公厅 国务院办公厅印发《关于进一步加强科研诚信建设的若干意见》

http://most.gov.cn/mostinfo/xinxifenlei/fgzc/gfxwj/gfxwj2018/201805/t20180531_139731.htm

（通讯作者：周红玲 E-mail：hongling@gigasciencejournal.com）
